# Autoimmune Hypothyroidism As a Predictor of Mortality in Chronic Hypersensitivity Pneumonitis

**DOI:** 10.3389/fmed.2017.00170

**Published:** 2017-10-16

**Authors:** Ayodeji Adegunsoye, Justin M. Oldham, Aliya N. Husain, Lena Chen, Scully Hsu, Steven Montner, Jonathan H. Chung, Rekha Vij, Imre Noth, Mary E. Strek

**Affiliations:** ^1^Department of Medicine, Section of Pulmonary and Critical Care Medicine, University of Chicago, Chicago, IL, United States; ^2^Department of Medicine, Division of Pulmonary, Critical Care and Sleep Medicine, University of California, Davis, Davis, CA, United States; ^3^Department of Pathology, University of Chicago, Chicago, IL, United States; ^4^Department of Radiology, University of Chicago, Chicago, IL, United States

**Keywords:** autoimmunity, hypothyroidism, hypersensitivity pneumonitis, extrinsic allergic alveolitis, pulmonary fibrosis

## Abstract

**Background:**

Chronic hypersensitivity pneumonitis (CHP) is a fibrotic parenchymal lung disease that occurs when inhalation of environmental antigens leads to immune dysregulation. Autoimmune features have recently been identified as potentially important among patients with CHP. However, the relationship between hypothyroidism (HT) and CHP is unknown. In this study, we investigate the prevalence and impact of HT among patients with CHP.

**Methods:**

We conducted a retrospective, case–control analysis. We identified 121 patients at the University of Chicago Interstitial Lung Disease Center with a multidisciplinary diagnosis of CHP. These patients were matched 3:1 according to age, sex, and race to 363 control subjects with asthma from 2006 to 2015. We analyzed demographics, clinical characteristics, and survival between both groups and assessed the relationship of HT with CHP. Survival analysis was performed using Cox proportional hazards modeling.

**Results:**

Patients with CHP had higher prevalence of HT (25.6%, *n* = 31) compared to controls (10.7%, *n* = 39; OR, 2.86; 95% CI, 1.62–4.99; *P* < 0.0001). Compared to CHP alone, patients with CHP/HT were more likely to be female (80.6 vs 51.1%, *P* = 0.004), have increased incidence of autoimmune disease (19.4 vs 3.3%, *P* = 0.009), antinuclear antibody seropositivity (80.6 vs 57.0%, *P* = 0.019), and higher TSH levels (4.0 vs 1.9 mIU/L, *P* < 0.0001). HT was a significant independent predictor of mortality among CHP patients with seropositive ANA (HR, 3.39; 95% CI, 1.31–8.80; *P* = 0.012).

**Conclusion:**

HT is common in patients with CHP and may carry prognostic significance in patients with features of autoimmunity. Further research exploring common pathogenic pathways between autoimmune HT and CHP may illuminate the association of HT with survival.

## Summary at a Glance

The prevalence of hypothyroidism and its prognostic impact in chronic hypersensitivity pneumonitis (CHP) are unknown. This study demonstrates a threefold increase in HT compared to the general population and shows that, in patients with CHP and antinuclear antibody (ANA) seropositivity, HT independently predicts mortality, providing insight into its influence on prognosis.

## Introduction

Hypersensitivity pneumonitis (HP) is a diffuse interstitial lung disease (ILD) resulting from a dysregulated immune response to inhaled environmental antigens. Due to complex immunologic interactions, up to one-fifth of exposed individuals may develop HP (1–3). When HP results in pulmonary fibrosis, also known as chronic HP (CHP), survival is worse (4). The heterogeneous nature of these immune responses and variation in clinical course suggests, however, that multiple pathways may be involved in disease initiation and progression. Several underlying mechanisms for autoimmune diseases, such as shared genetic pathways, tissue microchimerism, and exposure to environmental antigens, have been implicated in the pathogenesis of CHP (5–8). We recently showed that features of autoimmunity are common among patients with CHP (9) and that HT, an autoimmune process characterized by autoantibody-mediated thyroid inflammation and destruction, is prevalent among patients with idiopathic pulmonary fibrosis (IPF) (10).

Hypothyroidism affects up to 9% of women and 1–2% of men in the general population (11, 12). HT occurs predominantly in developed nations as the sequelae of an underlying autoimmune process, though a minority of cases may be congenital, postpartum, or result of treatment with medications such as glucocorticoids (12–17). Systemic corticosteroid medications, used frequently in the treatment of patients with chronic HP, may also cause HT through suppression of thyroid-stimulating hormone (TSH) (18).

The prevalence and impact of HT in CHP is unknown. We hypothesized that (1) HT is more common in patients with CHP than in matched control subjects and (2) the increased prevalence of HT impacts survival in CHP and is associated with underlying autoimmune processes and not glucocorticoid therapy.

## Materials and Methods

### Study Population

A retrospective, case–control analysis was performed at the University of Chicago Hospitals, with approval of our Institutional Review Board (IRB16-1235). The University of Chicago ILD registry was screened for patients with CHP followed from 2006 to 2015. CHP cases were matched 3:1 according to age, sex, and race to individuals with a diagnosis of asthma followed at the University of Chicago during the same time period.

The diagnosis of CHP was determined through multidisciplinary review involving pulmonologists, thoracic radiologists, and pathologists according to ATS criteria as previously described (9, 19). Patients with other forms of ILD, including IPF and interstitial pneumonia with autoimmune features (IPAF), were excluded. Eligible controls were all individuals with asthma who had been evaluated at the University of Chicago from 2006 to 2015. Patients with an International Classification of Diseases, Ninth Revision code for asthma were systematically identified by the University of Chicago Center for Research Informatics and included in the matching algorithm. Using a random-number generator, we selected three control individuals per case, frequency matched by age, sex, and race/ethnicity. If race/ethnicity information was missing, the selected control was discarded and the next randomly selected eligible control with complete race/ethnicity information was chosen.

All data were extracted retrospectively from the electronic medical record using the initial clinic visit. These data included demographic information (age, race/ethnicity, sex), patient-reported medical/surgical history [HT, gastroesophageal reflux (GER), diabetes mellitus (DM), coronary artery disease (CAD), tobacco use, hyperthyroidism, thyroid ablation, thyroidectomy], environmental antigen exposure history (avian, mold, other, unknown), patient-reported medications [thyroid replacement, GER and statin therapy, lithium, amiodarone, systemic corticosteroids, radioactive iodine (RAI) history], physical examination findings [body mass index (BMI), clubbing, crackles], laboratory studies (ANA with staining pattern, rheumatoid factors, anticitrullinated protein antibody, myositis-specific antibodies, antineutrophil cytoplasmic antibody, anti-Ro/SSA antibody, anti-La/SSB antibody, anti-Scl-70 antibody, aldolase, TSH, and free thyroxine), diagnostic studies [high-resolution CT (HRCT) scan, surgical lung biopsy (SLB), pulmonary function testing, including FVC, FEV1, and percent predicted diffusion capacity of the lung for carbon monoxide (DLco)] and documented diagnosis of a defined autoimmune disease (Sjogren’s disease, scleroderma, systemic lupus erythematosus, idiopathic inflammatory myopathy, rheumatoid arthritis, and ulcerative colitis). Chronic glucocorticoid therapy was recorded when a patient reported a history of prednisone use equivalent to 5 mg daily or higher for 4 or more weeks (20). HT was recorded when a patient reported a history of HT, was using thyroid replacement therapy, and did not report a previous history of thyroidectomy or RAI ablation. Exploratory analysis was performed among the CHP cases to evaluate any associations between HT, serum TSH, glucocorticoid use, and underlying autoimmune processes. No patients reported the use of lithium, amiodarone, or interferon-gamma, which are known to alter thyroid function. No patients were immediately postpartum or endorsed a history of congenital HT.

### Statistical Analysis

Continuous variables are reported as means with SD and are compared using a two-tailed Student’s *t*-test. Categorical variables are reported as counts and percentages and were compared using the chi-squared test or Fisher exact test, as appropriate. Conditional logistic regression was performed to compare the proportion of HT between cases and control subjects. Survival analysis was performed using univariate and multivariable Cox regression together with the unadjusted log-rank test and was plotted using the Kaplan–Meier survival estimator. Survival time was defined as time from CHP diagnosis to death, transplant, loss-to-follow-up, or end of study period. Survival time was censored on April 30th, 2015 or at the time a patient underwent lung transplant or was lost to follow-up. All statistical analyses were performed using Stata 14 (StataCorp LP).

## Results

Of 161 individuals initially identified with a diagnosis of HP based on *International Classification of Diseases, Ninth Revision code* (Figure S1 in Supplementary Material), 121 were diagnosed with HP after multidisciplinary review, according to the 2013 American Thoracic Society/European Respiratory Society guidelines (19). Of those failing to meet the established guidelines for diagnosis of HP, three were missing relevant clinical information needed to verify the diagnosis: HRCT or SLB; 34 were given a diagnosis of an alternative ILD. Patients who exhibited clinical, radiographic, and pathologic features of HP but had antecedent history of chemotherapy (*n* = 3) were excluded from the analysis leaving 121 cases for the primary analysis. Of these 121 cases, 75 (62%) had undergone SLB, which demonstrated histopathologic features of HP, whereas the remainder demonstrated positive serum precipitins to specific antigens, lymphocytosis on bronchoalveolar lavage or compatible HRCT abnormalities that were consistent with their exposure history and clinical features of HP. All patients demonstrated HRCT or SLB features of fibrosis.

A comparison of baseline characteristics between cases and controls is shown in Table 1. Groups were similar with regard to age (65.1 vs 66.4 years, respectively), female sex (58.7 vs 58.1%), and non-Hispanic Caucasian race/ethnicity (83.5 vs 81.0%), as specified by the study design. Compared with controls, cases had a higher BMI (32.4 vs 30.0, *P* = 0.005), higher prevalence of ever-smokers (59 vs 46%, *P* = 0.016), and a higher frequency of documented glucocorticoid use (77.7 vs 48.2%, *P* < 0.001; respectively). There were no significant differences in the prevalence of DM between case patients and control subjects (19.0 vs 22.3%, respectively).

**Table 1:** Baseline characteristics.

Characteristic	CHP case patients (*n* = 121)	Control subjects with asthma (*n* = 363)	*P*-value
Age, years	65.1 ± 10.9	66.4 ± 10.9	0.278
Female	71 (58.7)	211 (58.1)	0.915
Race/ethnicity			0.892
White	101 (83.5)	294 (81.0)	0.542
Black	7 (5.8)	27 (7.4)	1.000
Hispanic	10 (8.3)	30 (8.3)	0.538
Asian	3 (2.5)	12 (3.3)	0.771
Ever smoker	71 (58.7)	167 (46.0)	**0.016**
Diabetes mellitus	23 (19.0)	81 (22.3)	0.443
BMI	32.4 ± 8.08	30.0 ± 7.9	**0.005**
Prior systemic glucocorticoid use	94 (77.7)	175 (48.2)	**<0.001**

*Bold indicates statistical significance at P < 0.05*.

When comparing the proportion of patients with HT between cases and controls (Table 2), HT was identified in 31 (25.6%) cases and 39 (10.7%) controls (OR, 2.86; 95% CI, 1.62–4.99; *P* < 0.0001). HT was identified in 5% of male cases compared with 2% of male controls and 21% of female cases compared with 9% of female controls. After adjustment for variables previously associated with HP, HT, or both, including BMI (21, 22), smoking history (7, 23), DM (24, 25), and corticosteroid use (16, 17, 26), HT remained significantly associated with CHP (OR, 2.39; 95% CI, 1.36–4.20; *P* = 0.002).

**Table 2:** Hypothyroidism (HT) and chronic hypersensitivity pneumonitis (CHP) risk.

Characteristic	CHP case patients (*n* = 121)	Control subjects with asthma (*n* = 363)	Unadjusted results			Adjusted results^a^		
			OR	*P*-value	95% CI	OR	*P*-value	95% CI
HT	31 (25.6)	39 (10.7)	2.86	**0.0001**	1.62–4.99	2.39	**0.002**	1.36–4.20
Male	6 (5.0)	6 (1.7)						
Female	25 (20.7)	33 (9.1)						

*^a^Adjusted for BMI, smoking history, diabetes mellitus, and glucocorticoid use*.

*Bold indicates statistical significance at P < 0.05*.

We then stratified patients with CHP based on HT status (Table 3). The proportion of females in the CHP/HT subgroup was greater than that of the CHP subgroup (80.6 vs 51.1%, *P* = 0.004). Those with CHP/HT were also found to have significantly greater incidence of autoimmune disease (19.4 vs 3.3%, *P* = 0.009), ANA seropositivity (80.6 vs 57.0%, *P* = 0.019); rheumatoid factor/anticitrullinated protein antibody seropositivity (9.7 vs 1.1%, *P* = 0.021); >1 autoantibody seropositivity (19.4 vs 5.6%, *P* = 0.027); higher TSH levels (4.0 vs 1.9 mIU/L, *P* < 0.0001) and radiographic mosaic attenuation (96.8 vs 81.1%, *P* = 0.035) compared with those with CHP alone. No significant differences were observed between groups with respect to the following: age; race/ethnicity; BMI; crackles; clubbing; smoking history; GER; DM; the use of glucocorticoid therapy; FVC% predicted; DLCO% predicted; requirement for oxygen therapy, 6-min walk distance; radiographic ground glass opacities, traction bronchiectasis or honeycomb pattern; histopathologic presence of poorly formed granulomas, lymphoplasmacytic infiltration or germinal centers, UIP pattern; gender, age, physiology (GAP) stage (27); or lung transplant.

**Table 3:** Baseline characteristics of hypothyroid cohort among patients with hypersensitivity pneumonitis.

Characteristic	CHP/HT (*n* = 31)	CHP only (*n* = 90)	*P*-value
Age, years mean (±SD)	65.6 ± 8.4	65 ± 11.7	0.795
Female, *n* (%)	25 (80.6)	46 (51.1)	**0.004**
**Race/ethnicity**			
White, *n* (%)	28 (90.3)	73 (81.1)	0.277
Black, *n* (%)	0 (0.0)	7 (7.8)	0.189
Hispanic, *n* (%)	1 (3.2)	9 (10.0)	0.450
Asian, *n* (%)	2 (6.5)	1 (1.1)	0.161
BMI, mean (±SD)	33.3 ± 9.1	32.1 ± 7.7	0.485
Crackles, *n* (%)	26 (83.9)	77 (85.6)	0.777
Clubbing, *n* (%)	5 (16.1)	24 (26.7)	0.330
Ever smoker, *n* (%)	18 (58.1)	53 (58.9)	0.936
**Antigen exposure**			
Avian, *n* (%)	13 (41.9)	44 (48.9)	0.504
Mold, *n* (%)	10 (32.3)	25 (27.8)	0.635
Hot tub, *n* (%)	0 (0.0)	3 (3.3)	0.569
Unknown, *n* (%)	9 (29.0)	29 (32.2)	0.825
Gastroesophageal reflux, *n* (%)	21 (67.7)	50 (55.6)	0.235
Diabetes mellitus, *n* (%)	8 (25.8)	15 (16.7)	0.263
Coronary artery disease, *n* (%)	8 (25.8)	17 (18.9)	0.412
Any prior glucocorticoid use, *n* (%)	27 (87.1)	67 (74.4)	0.211
Chronic glucocorticoid therapy, *n* (%)	22 (71.0)	61 (67.8)	0.741
Autoimmune disease, *n* (%)	6 (19.4)	3 (3.3)	**0.009**
ANA seropositivity^a^, *n* (%)	25 (80.6)	49 (57.0)	**0.019**
RF or aCCP seropositivity^b^, *n* (%)	3 (9.7)	1 (1.1)	**0.021**
≥1 autoantibody, n (%)	6 (19.4)	5 (5.6)	**0.027**
TSH^c^ mIU/mL, mean (±SD)	4.0 ± 3.6	1.9 ± 1.5	**< 0.001**
FVC% predicted, mean (±SD)	64.1 ± 21.7	65.2 ± 17.9	0.779
DLCO% predicted, mean (±SD)	53.9 ± 27.0	54.8 ± 24.1	0.856
Oxygen therapy, *n* (%)	19 (61.3)	49 (54.4)	0.508
6MWT distance (feet)^d^, mean (±SD)	1,009.1 ± 411.3	1,140.7 ± 457.9	0.173
**HRCT features**			
Mosaic attenuation, *n* (%)	30 (96.8)	73 (81.1)	**0.035**
Ground glass opacities, *n* (%)	30 (96.8)	84 (93.3)	0.479
Traction bronchiectasis, *n* (%)	22 (71.0)	76 (84.4)	0.100
Radiographic honeycomb pattern, *n* (%)	11 (35.5)	40 (44.4)	0.384
**Histopathologic features^e^**			
Surgical lung biopsy obtained, *n* (%)	20 (64.5)	55 (61.1)	0.736
Poorly formed granulomas, *n* (%)	16 (80.0)	33 (60.0)	0.108
Lymphoplasmacytic infiltration/GC, *n* (%)	7 (35.0)	12 (21.8)	0.246
Honeycombing with UIP pattern, *n* (%)	8 (40.0)	27 (49.1)	0.485
**ILD-GAP index, mean (±SD)**			
0–1	8 (40.0)	18 (20.0)	0.497
2–3	9 (29.0)	29 (32.2)	0.741
4–5	9 (29.0)	30 (33.3)	0.659
>5	5 (16.1)	13 (14.4)	0.777
Lung transplant, *n* (%)	1 (3.2)	4 (4.4)	1.000

*^a^ANA data available for 117 patients; Positive ANA titer ≥1:160*.

*^b^RF positivity at >3× ULN*.

*^c^TSH data obtained in 86 patients (25 HT, 51 non-HT)*.

*^d^6MWT available for 115 pts*.

*^e^Surgical lung biopsy obtained in 75 patients*.

*Bold indicates statistical significance at P < 0.05*.

**Figure 1:**
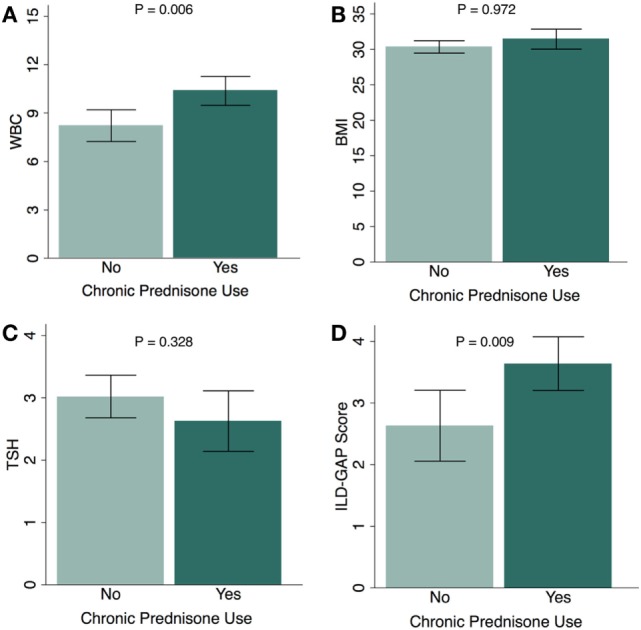
Chronic glucocorticoid use and physiologic parameters in chronic hypersensitivity pneumonitis (CHP) cohort. Relationship between chronic glucocorticoid therapy and **(A)** white blood cell count (WBC); **(B)** body mass index (BMI); **(C)** thyroid-stimulating hormone (TSH); and **(D)** ILD-GAP Score in patients with CHP. (Patients receiving chronic glucocorticoid therapy, *N* = 83; patients not receiving chronic glucocorticoid therapy, *N* = 38.) Exception for number of patients: WBC (*n* = 111), TSH (*n* = 86). Results are shown as mean ± SD.

When sub-stratified based on chronic glucocorticoid therapy, the subgroup of CHP patients who had received chronic glucocorticoids had a higher white blood cell count (*P* = 0.006) (Figure 1A), similar BMI (*P* = 0.972) (Figure 1B), similar TSH (*P* = 0.328) (Figure 1C), and higher ILD-GAP score (*P* = 0.009) (Figure 1D). When comparing the proportion of patients who had received chronic glucocorticoid therapy among CHP patients with HT and CHP patients without HT, there was no significant association between HT and chronic glucocorticoid therapy in univariate analysis (OR, 1.16; 95% CI, 0.44–3.24; *P* = 0.741) or after multivariate adjustment (OR, 1.12; 95% CI, 0.45–2.79; *P* = 0.810) (Table S1 in Supplementary Material).

**Figure 2:**
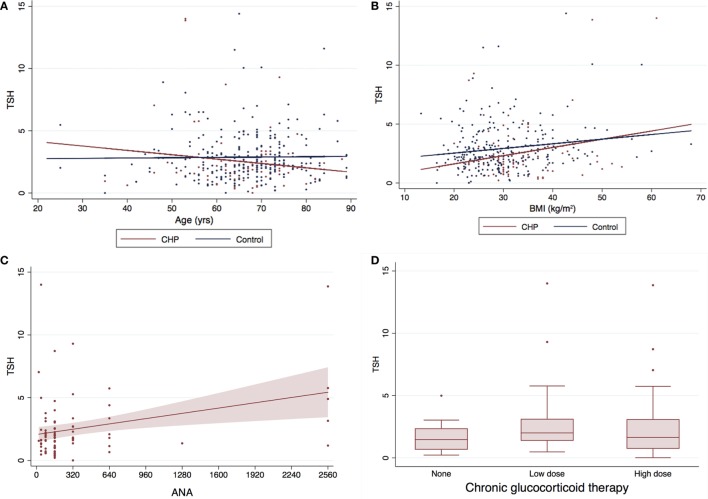
Serum TSH levels and baseline characteristics of CHP cohort*. The mean TSH for the CHP cohort was marginally lower (0.53 mIU/L) than the control population (2.52 ± 2.47 vs 3.05 ± 2.78; *P* = 0.121). There was no correlation between **(A)** TSH and age (*R* = −0.036, *P* = 0.502) or **(B)** TSH and body mass index (BMI) (*R* = 0.086, *P* = 0.115). Serum TSH levels **(C)** correlated positively with antinuclear antibody (ANA) titers (*R* = 0.2997; *P* = 0.0043), and **(D)** did not differ with/without glucocorticoid therapy (*P* = 0.497). Panel **(C)** only includes patients with CHP and no controls. *Two data points with TSH >20 included in the analysis were not depicted in the graph above for the purpose for clarity. TSH, thyroid-stimulating hormone; CHP, chronic hypersensitivity pneumonitis.

Eighty-six patients had serum TSH levels available for analysis. There was no difference in TSH levels between this subgroup of CHP patients and control subjects (2.52 ± 2.47 vs 3.05 ± 2.78; *P* = 0.121) (Figure 2). TSH did not correlate with an increase in age (*R* = −0.036, *P* = 0.502) (Figure 2A), or BMI (*R* = 0.086, *P* = 0.115) (Figure 2B). However, analysis of the CHP cohort revealed a positive correlation of serum TSH levels with ANA titers (*R* = 0.2997; *P* = 0.0043) (Figure 2C). Lower TSH levels were associated with traction bronchiectasis on chest imaging (*P* = 0.04). TSH levels did not differ between patients who had received chronic glucocorticoid therapy and those who had not (*P* = 0.497) (Figure 2D).

**Figure 3:**
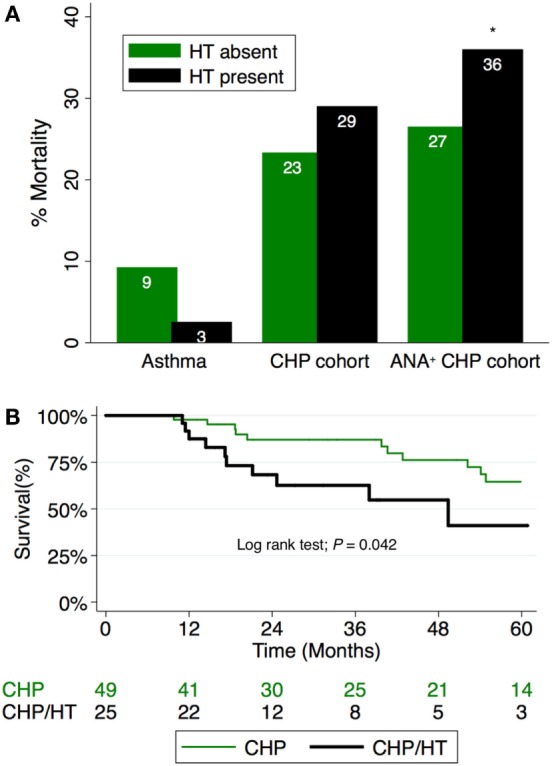
**(A)** Percentage mortality in CHP cohort compared to controls. Mortality associated with hypothyroidism (HT) in CHP is worsened in patients with positive antinuclear antibody (ANA) titers (interaction term *P*-value; *P* = 0.024). **(B)** Survival among patients with CHP and positive ANA stratified by HT status. Patients with combined HT and CHP demonstrate significantly reduced survival time compared with those with CHP alone (log-rank test; *P* = 0.042). CHP, chronic hypersensitivity pneumonitis; HT, hypothyroidism.

Although the presence of HT did not independently predict survival in the entire CHP cohort (HR, 2.14; 95% CI, 0.96–4.78; *P* = 0.062), survival analysis of the CHP cohort demonstrated significant interaction between HT and positive ANA (interaction term *P*-value; *P* = 0.024). When unadjusted survival analysis of CHP patients who had a positive ANA was performed, those with HT demonstrated significantly shorter survival compared with those without HT (log-rank test *P* = 0.04) (Figure 3). To identify predictors of mortality in the cohort of CHP patients who had a positive ANA, we performed univariate and multivariable Cox regression analysis (Table 4). Univariate analysis revealed HT to be a significant predictor of mortality [hazard ratio (HR), 2.35; 95% CI, 1.01–5.50; *P* = 0.048]. Each increase in GAP stage was also demonstrated to predict mortality (HR, 1.64; 95% CI, 1.26–2.14; *P* = 0.001). While the predictive value of BMI and chronic glucocorticoid therapy trended towards statistical significance (HR, 0.94; 95% CI, 0.89–1.00; *P* = 0.069 and HR, 2.64; 95% CI, 0.90–7.77; *P* = 0.078, respectively), other variables including radiographic honeycomb pattern of fibrosis (28, 29) and antigen identification (7) were not predictive of survival in univariate analysis. Inclusion of these variables in a multivariable model, along with BMI and race/ethnicity, demonstrated that HT remained significantly predictive of mortality (HR, 3.39; 95% CI, 1.31–8.80; *P* = 0.012), as did incremental change in the GAP stage (HR, 1.54; 95% CI, 1.10–2.14; *P* = 0.011).

**Table 4:** Predictors of mortality in chronic hypersensitivity pneumonitis (CHP).

Characteristic	Unadjusted results			Adjusted results^a^		
	HR	*P*-value	95% CI	HR	*P*-value	95% CI
**CHP cohort (***n*** = 121)**						
Hypothyroidism (HT)	1.34	0.444	0.63–2.82	2.14	0.062	0.96–4.78
ILD-GAP score	1.69	**<0.001**	1.39–2.06	1.59	**<0.001**	1.27–2.00
BMI	0.94	0.023	0.89–0.99	0.95	0.083	0.90–1.01
Chronic glucocorticoid use	3.02	0.023	1.16–7.86	1.53	0.412	0.55–4.26
**CHP cohort with positive ANA (***n*** = 74)**						
HT	2.35	**0.048**	1.01–5.50	3.39	**0.012**	1.31–8.80
ILD-GAP score	1.64	**<0.001**	1.26–2.14	1.54	**0.011**	1.10–2.14
BMI	0.94	0.069	0.89–1.00	0.95	0.196	0.89–1.02
Chronic glucocorticoid use	2.64	0.078	0.90–7.77	1.87	0.324	0.54–6.52

*^a^Adjusted for race/ethnicity, BMI, radiographic honeycomb pattern, identified antigen, ILD-GAP score, hypothyroidism and chronic glucocorticoid use*.

*ANA, antinuclear antibody titer; positive ANA ≥1:160*.

*Bold indicates statistical significance at P < 0.05*.

## Discussion

We report, for the first time, an association between HT and CHP. Compared to the general population in which HT affects up to 9% of women and 2% of men, 21% of the women, and 5% of the men in our CHP cohort had HT. Our study showed that the proportion of CHP/HT cases also exceeded that of matched control subjects with asthma. We found that the presence of HT was associated with autoimmune serologies. We also show that in those subjects with CHP and a positive ANA, HT was an independent predictor of mortality.

Although specific reasons for HT in our CHP cohort cannot be determined in this retrospective analysis, several factors previously associated with autoimmune HT are prevalent in our CHP cohort. Exposure to environmentally inhaled antigens, a characteristic feature of CHP, has been associated with development of autoimmune HT (8, 30). The majority of our CHP cohort were smokers or had identifiable exposure to inhaled environmental antigens. In addition, X-chromosome inactivation has been described as an important contributor to the increased female risk for autoimmune HT and carries prognostic value in affected patients (30–32). In our study, an overwhelming majority of HT subjects were female. The increased prevalence of environmental exposure in our cohort supports postulations by other investigators that link differences in individual environmental exposure to gender differences in prevalence of CHP (28, 33, 34). In our study, the greater female predominance and a high rate of exposure to identifiable environmentally inhaled antigens could have contributed to the increased prevalence of HT in our CHP cohort.

Further, HT has been associated with several autoimmune diseases such as Sjogren syndrome, systemic lupus erythematosus, and rheumatoid arthritis (35–37). This is consistent with data from our cohort which demonstrates an increased prevalence of autoimmune diseases in the CHP subset with HT. This suggests that shared biological pathways may contribute to development of HT in CHP patients with autoimmune diseases. The female predominance in our CHP/HT subpopulation may also reflect the increased prevalence of autoimmune HT in women. As expected, those with CHP/HT demonstrated a significant increase in the incidence of autoimmune disease and serologies concurrent with an increase in their TSH levels.

In our study, the proportion of control subjects with HT was notably higher than that of the general population. Thyroid hormones are thought to influence the inflammatory component of asthma possibly through enhancing IgE production (38). A large population study by Goldacre et al. suggested a positive association of asthma with HT (39). Harrison et al. measured serum thyroxine values and specific airway conductance as an index of beta-adrenergic responsiveness in hypothyroid patients (40). They demonstrated an inverse relationship between airway beta-adrenergic responsiveness and the level of thyroid function. Hemminki et al. also demonstrated an increased risk for HT in obese individuals (21), which constituted a significant proportion of subjects with asthma in our control population. We explored this association in our control population but found that the proportion of HT did not differ among obese and non-obese subjects with asthma (data not shown).

Our findings add to an increasing body of recent evidence that links HT with greater susceptibility to lung injury through mechanisms that involve epithelial cell apoptosis and TGF-B signaling. We have previously shown that HT is common in patients with IPF, and independently predicted mortality (10). Alonso-Merino et al. demonstrated the ability of the thyroid hormone triiodothyronine to antagonize fibrotic processes *in vivo* through inhibition of TGF-β/SMAD-dependent transcriptional activation (41). In their study, they showed the potential therapeutic (anti-inflammatory and anti-fibrotic) effects of triiodothyronine in experimental models of ventilator-induced lung injury, skin, and hepatic fibrosis. Their results suggest that binding of triiodothyronine to its nuclear receptors could be beneficial in blocking progression of pulmonary fibrosis. Similarly, Barca-Mayo et al. showed that increase in lung deiodinase type-2, a critical mediator of thyroid hormone metabolism, protects against ventilator-induced lung injury in mouse models of functional HT (42). They found that treatment with triiodothyronine reversed the increased chemokine and cytokine inflammatory profiles within the lungs. Taken together, these findings might represent a plausible mechanistic explanation for the increased mortality observed in CHP/HT patients, and adequate repletion of the deficient thyroid hormones holds potential therapeutic appeal in decreasing progression of pulmonary fibrosis and mortality.

Thyroid transcription factor-1 (TTF-1) controls the expression of select genes in thyroid and lung tissue, and optimal levels are essential to maintain thyroid and lung function (43). Mutations in the gene encoding TTF-1, NKX2-1, have been associated with development of ILD and pulmonary fibrosis (44). Genetic factors resulting in immune dysregulation have also been associated with autoimmune HT, including polymorphisms in the genes for human leukocyte antigen (HLA) and cytotoxic T-lymphocyte antigen 4 (CTLA-4). HLA haplotypes, such as HLA-DRB1/3, HLA-DQA1, HLA-DQB1, and HLA-DPB1, are present in persons with autoimmune HT (45, 46). Recent studies demonstrate an increased prevalence of these same HLA gene polymorphisms in subjects with CHP from different genetic backgrounds (47, 48). Likewise, CTLA-4 has been implicated in the susceptibility to autoimmune HT, and may reduce inflammatory lung disease in murine models of CHP (49–52). Our findings support these studies, which implicate common genetic pathways in the pathogenesis of CHP and autoimmune HT.

Our study was limited by several factors. First, our findings represent an association but do not infer causality due to the retrospective design of our investigation. Second, it was not possible to biochemically confirm autoimmune HT using anti-TPO or anti-thyroglobulin in the entire cohort because the diagnosis had been made several years before referral to our institution. Thus, we used a previously published algorithmic approach to the diagnosis of HT in ILD patients (10). Additionally, as the high prevalence of ANA seropositivity in autoimmune HT is well described (53–56), we elected to use ANA seropositivity as a marker of autoimmune HT in our CHP/HT cohort. Third, we used data from electronic medical records containing patient-reported medical history and medications. Data were collected as a part of clinical care, and not specifically for this study.

## Conclusion

We demonstrate that in patients with CHP, HT is a common finding and may have prognostic value in the subset of patients with a positive ANA. In a well-characterized cohort of subjects with CHP, HT was not associated with glucocorticoid use. As the role of the immune system is increasingly studied in the pathogenesis and progression of CHP, further research identifying common pathogenic pathways between autoimmune HT and CHP may elucidate the association of HT with survival.

## Ethics Statement

Patient consent was obtained for enrollment and participation in the University of Chicago ILD registry and the study was approved by the University of Chicago Institutional Review Board (IRB16-1235).

## Author Contributions

Conception and design—AA, JO, RV, IN, and MS; data acquisition—AA, JO, LC, SH, AH, SM, JC, RV, IN, and MS; data analysis and interpretation—AA, JO, RV, and MS; drafting of manuscript for important intellectual content—AA, JO, LC, SH, AH, SM, JC, RV, IN, and MS. All authors read and approved the final manuscript.

## Conflict of Interest Statement

The authors declare that the research was conducted in the absence of any commercial or financial relationships that could be construed as a potential conflict of interest.

## Abbreviations

BMI, body mass index; CAD, coronary artery disease; CHP, chronic HP; CTLA-4, cytotoxic T-lymphocyte antigen 4; DLco, diffusion capacity of the lung for carbon monoxide; DM, diabetes mellitus; GAP, gender age physiology; GER, gastroesophageal reflux; HRCT, high-resolution CT; HLA, human leukocyte antigen; HP, hypersensitivity pneumonitis; HT, hypothyroidism; ILD, interstitial lung disease; IPF, idiopathic pulmonary fibrosis; RAI, radioactive iodine; SLB, surgical lung biopsy; TSH, thyroid-stimulating hormone.
