# Supplementary material for: Autoimmune Hypothyroidism As a Predictor of Mortality in Chronic Hypersensitivity Pneumonitis
Source: Front Med (Lausanne). 2017 Oct 16;4:170. doi: 10.3389/fmed.2017.00170 (PMC5650730; doi:10.3389/fmed.2017.00170)
Supplement: Supplementary file 1 [file data_sheet_1.docx]

**E-Supplement**

| **Supplementary Table S1. Hypothyroidism and Chronic Glucocorticoid Risk among CHP cohort** | | | | | | | | | | | |
| --- | --- | --- | --- | --- | --- | --- | --- | --- | --- | --- | --- |
|  |  |  |  | |  | |  |  | |  |  |
| **Characteristic** | **CHP/HT**  **(n = 31)** | **CHP Alone**  **(n = 90)** | **Unadjusted Results** | | | | | **Adjusted Results*** | | | |
|  |  |  | **OR** | **P Value** | | **95% CI** | | **OR** | **P Value** | | **95% CI** |
| Chronic Glucocorticoid use | 22 (71.0) | 61 (67.8) | 1.16 | 0.741 | | 0.44 - 3.24 | | 1.12 | 0.810 | | 0.45 - 2.79 |
| Male | 3 (9.7) | 28 (31.1) |  |  | |  | |  |  | |  |
| Female | 19 (61.3) | 33 (36.7) |  |  | |  | |  |  | |  |
|  |  |  |  |  | |  | |  |  | |  |
| *Adjusted for BMI, smoking history, diabetes mellitus and gastroesophageal reflux | | | | | |  | |  |  | |  |

| **Supplementary Table S2. Antinuclear antibody (ANA) characteristics in patients with chronic hypersensitivity pneumonitis (CHP) and positive ANA** | | | | |
| --- | --- | --- | --- | --- |
|  |  |  |  |  |
| **Subjects** |  | **CHP/HT**  **(n = 25)** | **CHP only**  **(n = 49)** | **P value** |
| **ANA titer, n (%)** | |  |  |  |
| 1:160 |  | 14 (56) | 25 (51) | 0.685 |
| 1:320 |  | 5 (20 | 15 (31) | 0.331 |
| 1:640 |  | 3 (12) | 6 (12) | 0.976 |
| 1:1280 |  | 0 (0) | 1 (2) | 0.472 |
| 1:2560 |  | 3 (12) | 2 (4) | 0.199 |
| **ANA pattern*, n (%)** | |  |  |  |
| Nucleolar |  | 0 (0) | 5 (10) | 0.098 |
| Speckled |  | 15 (60) | 24 (49) | 0.369 |
| Homogenous | | 10 (40) | 20 (41) | 0.946 |
| * ANA pattern not documented in one patient. No patient had a centromere or diffuse pattern | | | | |


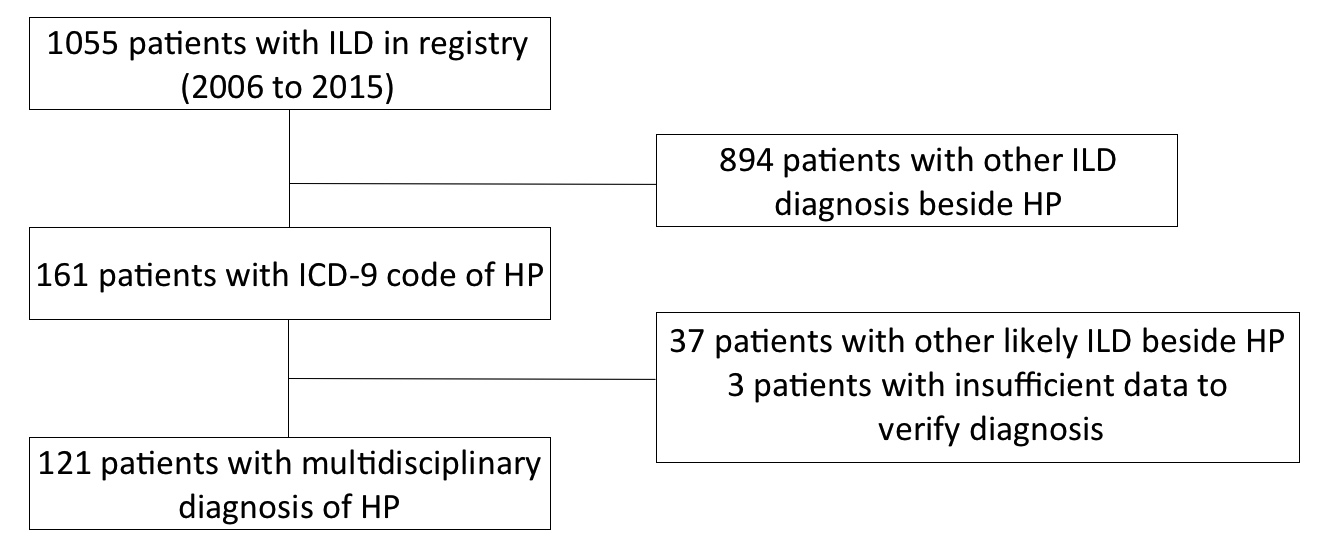


**Supplementary Figure S1. Consort diagram**
